# Creation of a PDMS Polymer Brush on SiO_2_-Based Nanoparticles by Surface-Initiated Ring-Opening Polymerization

**DOI:** 10.3390/polym12040787

**Published:** 2020-04-02

**Authors:** Karin Koch, Sven Geller, Kubilay Acar, Patricia Bach, Ekaterina Tsarenko, Annette Schmidt

**Affiliations:** Institut für Physikalische Chemie, Department Chemie, Universität zu Köln, 50923 Köln, Germany; karin.koch@uni-koeln.de (K.K.); sven-geller@web.de (S.G.); kacar@smail.uni-koeln.de (K.A.); pbach@uni-koeln.de (P.B.); carenkok96@mail.ru (E.T.)

**Keywords:** grafting-from, surface functionalization, cyclosiloxanes, ring–chain equilibrium

## Abstract

The incorporation of nanoparticles into soft matrices opens a broad spectrum of novel property combinations. However, one of the major challenges for these systems remains the compatibilization of particles with the surrounding matrix by proper surface functionalization. For silicon-based systems or liquid crystalline phases, polydimethylsiloxane (PDMS) brushes at the surface of particles increase the stability against particle agglomeration in such systems. Here, we report a novel approach for the functionalization of particles with a polysiloxane brush by surface-initiated ring-opening polymerization of a cyclosiloxane. For this purpose, surface hydroxy groups of silica and silica-coated hematite particles are used as initiators in combination with phosphazene bases as catalysts. The ring–chain equilibrium of a model-based solution polymerization is investigated in detail to find the appropriate reaction parameters. The corresponding molar masses are determined and compared by ^1^H-NMR and SEC measurements to confirm the underlying mechanism. In the resulting hybrid nanostructures, a covalently bound PDMS fraction is achieved up to 47 mass %.

## 1. Introduction

Soft hybrid nanocomposites based on both organic and inorganic components attract considerable attention since they open the pathway to combine fundamentally different functions and functionalities in one material, and thus enable the design of novel smart devices. By tailoring the properties of the utilized nanostructures, e.g., inorganic nanoparticles and organic or polymeric matrices), the material can be responsive to various magnetic, electric and mechanical stimuli. 

Strong coupling effects in these hybrid nanocomposites, material stability and reversibility thereby significantly depend on the stability of the particles against phase separation. Today, surface functionalization of inorganic nanoparticles is a common stabilization route and thus is a key for the fabrication of soft nanocomposites. Here, the surface modification with covalently bound polymer layers or brushes is a particularly successful compatibilization method when it comes to well-defined systems [1,2,3,4,5,6,7,8,9,10]. 

In general, the choice of the type of polymer for the particle compatibilization with its environment needs to be adapted to the final composite matrix. For hydrophobic materials, such as silicones or liquid crystalline phases, polydimethylsiloxane (PDMS) brushes created by a “grafting-to” process are shown to be suitable coatings to increase the stability of the system [7,11,12,13,14]. In contrast, end-tethered polymer brushes that are created by surface-initiated polymerization (SIP, “grafting-from”) can be much denser, and if properly controlled, of significant thickness. In a grafting-from process, linear polymer chains are generated by direct growth. SIP can be divided into surface-confined and non-confined processes, where apart from surface-attached polymers, free polymers are also formed, and are now reported for use in a wide range of monomers. The main mechanisms include controlled radical polymerization (styrene [15], methyl methacrylate [16]) and ring-opening polymerization (ε-caprolactone [10,17,18,19], p-dioxanone [8], lactide [2,20]). However, for the ring-opening polymerization of cyclosiloxanes, up to now, to our knowledge a SIP has not been reported yet, although it opens a wide range of future options for functional nanoparticle coatings. 

There are various pathways for the synthesis of PDMS, generally classified into step polymerization of end-functional oligosiloxanes and chain ring-opening polymerization of cyclosiloxanes [21,22]. A suitable mechanism for the generation of a PDMS brush by SIP has to fulfill a number of requirements. First of all, the pathway has to be free of branching or crosslinking side reactions, in particular the latter, which can lead to particle agglomeration. In addition, it is essential to possess an exclusive or at least competitive surface initiation process. Chain transfer reactions, if any, in addition, should not lead to a loss of end group control. These requirements are not in agreement with step-growth mechanisms as they possess only statistical end group control [23]. Further, for platinum-catalyzed hydrosilylation reactions, crosslinking side reactions are frequently reported [24]. As a feasible process for the surface-initiated polymerization towards polysiloxanes, anionic ring-opening (ROP) polymerization of cyclosiloxanes is a promising method, as it allows the creation of linear macromolecules with predefined end groups. 

The bulk- or solution-based ring-opening polymerization of cyclosiloxanes is well-investigated, and is generally performed under acidic or basic conditions. [21,25,26,27,28,29,30,31,32,33,34]. For cationic ROP, various strong protonic acids such as sulfuric acid, triflourmethane sulfonic acid [25,27,30] or Lewis acids such as ferric chloride or tris(pentafluorophenyl)borane [28,35] are employed together with a low molecular linear siloxane used and as chain-controllers leading to high molar masses with good reaction control. However, in order to be useful in an SI–ROP, a surface-attached chain controller would be required that is experimentally difficult to access due to the ease of condensation between different adjacent siloxane groups in confinement. In comparison, an anionic ROP mechanism catalyzed by strong inorganic or organic nucleophilic bases is better suited for the purpose. Usually, alkali metal hydroxides such as KOH, alcoholates, silonates or lithium organyls are used as initiators/catalysts. It is well known that, this way, high-molecular polymers with excellent end group control are accessible [31,36,37]. The polymerization rate strongly depends on the size of the cation [21,26]. However, the use of these initiators/catalysts involves problems like low solubility, long reaction times and the requirement for high temperatures [36,38]. Molenberg et al. [39,40] reported a fast and effective catalytic system for these monomers using the organophilic phosphazene bases developed by Schwesinger and Schlemper [41,42] as catalysts. Phosphazene bases in combination with alcohols form very soft and large cations with a great solubility in apolar solvents. Compared to other catalysts, this system polymerizes cyclosiloxanes fast and under very mild conditions [37,39,43]. For SI–ROP, it is useful that the end group control inherent to the mechanism allows initiation from surface-attached, deprotonated hydroxyl groups. Owing to the strong basicity of the basic form and the large size of the conjugate acid cation, phosphazene bases are identified as the most promising concept towards the SI–ROP of cyclosiloxanes. 

We here report a novel route towards the surface-initiated functionalization of SiO_2_-coated nanoparticles with PDMS brushes using octamethylcyclotetrasiloxane (M_4_) as a monomer. In this process, we employ inherent surface hydroxy groups as the initiator species, and a phosphazene base as catalyst. We investigate suitable reaction parameters in a model, solution-based polymerization and compare two different base catalysts, P_4_-*t*-Bu and P_2_-*t*-Bu. We show that surface hydroxy groups of silica (SiO_2_) and silica-coated hematite (SiO_2_@α-Fe_2_O_3_) particles in combination with phosphazene bases initiate the ROP of M_4_, and demonstrate how to modify the reaction conditions to increase the PDMS content.

## 2. Materials and Methods 

### 2.1. Chemicals

Iron (III) chloride hexahydrate (FeCl_3_∙6H_2_O, 99%), tetramethylammonium hydroxide (TMAH, 25% in water), 2,2-dimethyl-1-propanol (NOH, 99%), 1-tert-butyl-4,4,4-tris(dimethylamino)-2,2-bis[tris(dimethylamino)-phosphoranylidenamino]-2λ^5^,4λ^5^-catenadi(phosphazene) (P_4_-*t*-Bu, 0.8 M in hexane), 1-tert-butyl-2,2,4,4,4-pentakis(dimethylamino)-2λ^5^,4λ^5^-catenadi(phosphazene) (P_2_-*t*-Bu, 2 M in THF) and clorotrimethylsilane (CTMS, 98%) were purchased from Sigma Aldrich (Darmstadt, Germany). Octamethylcyclosiloxane (M_4_, 99%), tetraethylorthosilicate (TEOS, 99.9%) and ammonium hydroxide solution (NH_4_OH, 25 wt % in water) were obtained from ABCR (Karlsruhe, Germany). Sodium dihydrogen phosphate monohydrate (NaH_2_PO_4_∙H_2_O, 98%) was bought from Alfa Asear (Kandel, Germany). L-arginine (99%) was purchased from TCI. Toluene (HPLC) and calcium hydride (CaH_2_, 93%) were brought from Acros Organics (Geel, Belgium). Ethanol (HPLC), methanol (HPLC), acetone (HPLC), cyclohexane (HPLC), iron (III) nitrate nonahydrate (Fe(NO_3_)_3_, laboratory reagent grade) and technical nitric acid (HNO_3_, 65 wt % in water) were purchased from Fisher Scientific (Schwerte, Germany). Deuterated chloroform (CDCl_3_) was obtained from Eurisotop (Saarbrücken, Germany). Citric acid monohydrate (≥99.5%) was bought from Jungbunzlauer GmbH (Ladenburg, Germany). M_4_ was distilled from CaH_2_. Toluene distilled over CaCl_2_. Other chemicals were used as received.

### 2.2. Instrumentation

^1^H-NMR spectra were taken on a Bruker Advance III 499 spectrometer (Billerica, Massachusetts) in CDCl_3_ at 499.2 MHz. Size exclusion chromatography (SEC) was performed with toluene as eluent on an SEC system from *hs-GmbH* with a four-column system of MZ-Analysetechnik (Mainz, Germany) with MZ-gel SD plus as the stationary phase, using a RI2012 reflective index detector from Schambeck (Bad Honnef, Germany) and multi-angle static light scattering (MALLS) detector Dark 4 from Consenxus (Ober-Hilbersheim, Germany) with a wavelength of 420 nm The system was calibrated using polystyrene standards, and the initial results were recalculated using the universal calibration approach based on the Mark–Houwink method (*K*_PDMS_ = 8.28 × 10^−3^ mL∙g^−1^; α = 0.72, *K*_PS_ = 8.50 × 10^−3^ mL∙g^−1^; α = 0.74) [44,45]) Transmission electron microscopy (TEM) images were taken using a Zeiss LEO 912 Omega (Jena, Germany). Elemental analyses of the amount of nitrogen, carbon and hydrogen were observed on an Elementar Vario EL (Langenselbold, Germany). IR spectra were measured on a Shimadazu IR Affinity-1 FT-IR spectrometer (Duisburg, Germany) using the total reflectance technique (ATR). Thermogravimetric analysis (TGA) of carefully vacuum-dried samples were performed on Perkin Elmer STA 6000 (Rodgau, Germany). After the samples were equilibrated at 30 °C for 10 min, they were heated to 800 °C with a heating rate of 10 K∙min^−1^. Water contents of solutions and compounds were measured on a 756 KF Coulometer from Metrohm (Fliderstadt, Germany).

### 2.3. Synthetic Procedures

#### 2.3.1. ROP of M_4_ Initiated by NeOH

*General procedure*: Under an argon atmosphere, 500 µL of the monomer M_4_ was dissolved in the appropriate volume of toluene (see below). The reaction was started by adding the initiator NeOH and an equivalent amount of the catalyst solution (P_4_-*t*-Bu or P_2_-*t*-Bu in hexane). After 10 min of stirring at ambient temperature, the reaction was quenched with an excess of CTMS. The polymer was obtained by precipitation in ice cold MeOH for two times and centrifugation at 7000 rpm for 10 min. Volatile residues were removed by carefully drying in a vacuum at 90 °C.

*Series 1*: The molar monomer-to-initiator ratio was kept at *n*_M_/*n*_I_ = 25, and the initial monomer concentration was varied between [M_4_]_0_ = 0.45 mol∙L^−1^ and [M_4_]_0_ = 1.50 mol∙L^−1^. The reaction was performed with P_4_-*t*-Bu as catalyst.

*Series 2*: The molar monomer-to-initiator was varied between n_M_/n_I_ = 5 and *n*_M_/*n*_I_ = 50 and the monomer concentration was kept constant at [M_4_]_0_ = 0.96 mol∙L^−1^. The reactions were performed with both P_4_-*t*-Bu and P_2_-*t*-Bu as catalysts, respectively.

^1^H-NMR (499 MHz, CDCl_3_): δ [ppm] = 3.28 (s, 2H), 0.87 (s, 9H), 0.07 (s, Si–CH_3_). The molar mass was calculated using the integral for the Si–CH_3_ A_0.07ppm_, the molar masses of M_4_ and the deprotonated species of NeOHand Equation (1)

(1) $$M_{n,NMR}=\frac{3\;\cdot \;A_{0.07\mathrm{ppm}}}{8\;\cdot \;A_{0.87\mathrm{ppm}}}\;\cdot \;M_{M4}+M_{{\mathrm{NeO}}^{-}}\;$$

#### 2.3.2. Synthesis of SiO_2_ Nanoparticles

Silica (SiO_2_) nanoparticles were synthesized using a modified Stöber process [46]. 362 mg (2.08 mmol) L-(+)-Arginin was solved in 276 mL water and 18 mL cyclohexane. The mixture was heated to 60 °C and 22 mL (99.27 mmol) TEOS were added. After stirring for 20 h at 60 °C and cooling to room temperature, the organic phase was separated and the aqueous phase was reduced to ~30 mL under reduced pressure. The particles were precipitated with ~150 mL acetone and collected via centrifugation at 8500 rpm for 15 min. SiO_2_ particles were washed four times with ethanol. Finally, the particles were transferred to toluene.

#### 2.3.3. Synthesis of SiO_2_@Fe_2_O_3_ Spindle-Shaped Nanoparticles

Spindle-like hematite (Fe_2_O_3_) particles were obtained via the hydrothermal method based on the synthetic route of Ozaki et al. [47]. In a three-necked flask with a reflux condenser, 1 L of distilled water was heated to 100 °C. After the water reached 100 °C, first 0.07176 g NaH_2_PO_4_∙H_2_O in 120 mL water and then 7.015 g FeCl_3_∙6H_2_O dissolved in 150 mL water was added to the reaction mixture. The solution was stirred under reflux for 72 h. After cooling to room temperature, the particles were collected via centrifugation (7000 rpm, 15 min) and washed with water five times. Finally, the particles were dispersed in water. The particles were electrostatically stabilized with citric acid following the protocol of Wagner et al. [48]. To a 35 mL solution of α-Fe_2_O_3_ in water, 30 mL of 2 M HNO_3_ was added and stirred 5 min at room temperature. Afterwards, 30 mL of an aqueous solution of Fe(NO_3_)_3_ (0.35 M) was added and the reaction was stirred to reflux for 1 h. After cooling to room temperature, the particles were collected via centrifugation (7000 rpm, 15 min) and washed one time with 2 M HNO_3_ and three times with water. To the resulting dispersion of Fe_2_O_3_ in water, 0.1 M citric acid solution was added dropwise until the particles precipitate, followed by the addition of tetramethylammonium hydroxide to set the pH to 8–9. Finally, the particles were washed two times with water.

Core-shell particles SiO_2_@Fe_2_O_3_ were synthesized based on the routes of Philipse [49] and Wagner [48]. One gram of Fe_2_O_3_ particles was dispersed in a mixture of 167 mL water, 667 mL EtOH and 21 mL NH_4_OH-solution. The mixture was treated in the ultrasonic bath while 914 µL TEOS were added in two aliquots with a time delay of 10 min between the aliquot. After a further 10 min in the ultrasonic bath, the reaction was allowed to stir overnight at room temperature. The particles were collected via centrifugation (7000 rpm, 15 min) and washed five times with EtOH. Finally, the particles were transferred to toluene by collecting the particles via centrifugation (7000 rpm, 15 min) and dispersing into toluene.

#### 2.3.4. SI–ROP of M_4_

*General procedure*: Under argon atmosphere, 50 mg of SiO_2_ or SiO_2_@Fe_2_O_3_ particles dispersed in the appropriate volume of toluene were mixed with P_4_-*t*-Bu. After adding the respective amount of monomer M_4_, the mixture was allowed to stir for at least 24 h and quenched with an excess of CTMS. The particles were then collected via centrifugation (8500 rpm, 10 min), washed six times with toluene and dried in vacuo. The supernatant of each washing process was collected and precipitated into cold MeOH to quantify and analyze free PDMS. The used amounts of reactants for the different reaction series were described in the following.

*Series 3*: 50 milligrams of SiO_2_ were dispersed in varying amounts of toluene to reach a final monomer concentration [M_4_]_0_ between 0.57 mol∙L^−1^ and 2.57 mol∙L^−1^. The monomer-to-initiator ratio was kept constant using 607 µL of M_4_ and 28 µL P_4_-*t*-Bu-solution.

*Series 4*: 50 milligrams of SiO_2_@Fe_2_O_3_ were dispersed in varying amounts of toluene to reach a final monomer concentration [M_4_]_0_ between 0.57 mol∙L^−1^ and 2.57 mol∙L^−1^. The monomer-to-initiator ratio was kept constant by using a constant amount of 607 µL of M_4_ and 8 µL P_4_-*t*-Bu-solution.

*Series 5*: 50 milligrams of SiO_2_ were dispersed in 820 µL toluene and mixed with a 28 µL P_4_-*t*-Bu solution. The amount of M_4_ was varied to reach a final monomer concentration [M_4_]_0_ between 0.45 mol∙L^−1^ and 1.49 mol∙L^−1^.

*Series 6*: 50 milligrams of SiO_2_@Fe_2_O_3_ were dispersed in 230 µL toluene and mixed with an 8 µL P_4_-*t*-Bu solution. The amount of M_4_ was varied to reach final monomer concentrations between [M_4_]_0_ = 0.45 mol∙L^−1^ and [M_4_]_0_ = 1.49 mol∙L^−1^.

*Series 7*: For the kinetic investigation, 400 mg SiO_2_ particles were dispersed in 6.5 mL toluene and mixed with 226 µL P_4_-*t*-Bu solution and 5.6 mL M_4_. After appropriate periods of time samples were taken and immediately introduced to a vial containing toluene and an excess of CTMS.

## 3. Results

### 3.1. Parameter Evaluation in Model Solution-Based Polymerization

For the formation of a well-defined polymer brush on the surface of nanoparticles or flat interfaces, the surface-initiated polymerization suitable grafting-from process is a powerful tool. The process involves the provision of appropriate surface-attached functional groups that serve as initiators, and a catalyst with appropriate selectivity. Further, the polymerization mechanism should possess the appropriate characteristics in order to allow proper control of the process. In particular, it is essential to exclude side reactions that would lead to crosslinking and thus imperfect surface brushes. In addition, it is desirable to understand and control the relation between surface-bound and free polymer, in cases that the latter cannot be excluded.

**Scheme 1:**
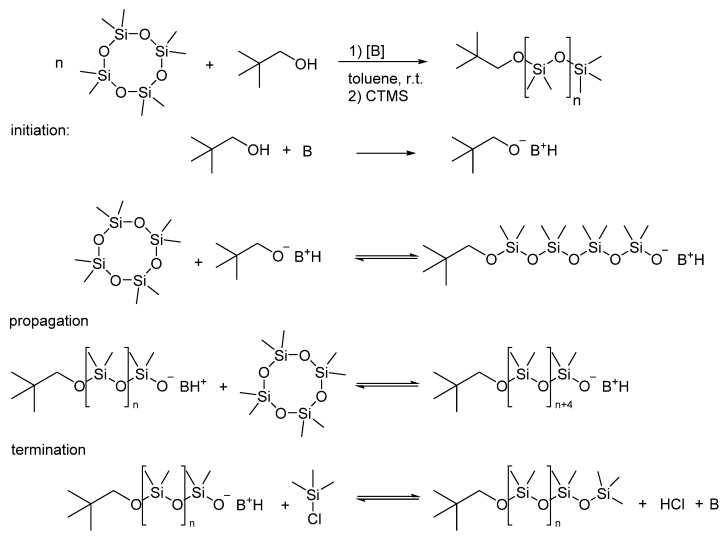
Mechanism of the ring-opening (ROP) of M_4_ initiated by NeOH and phosphazene base.

In the case of poly(dimethylsiloxane) and related polymers, the ring-opening polymerization of cyclosiloxanes is principally a suitable mechanism, as it enables a chain-type polymerization in the absence of branching side reactions. Suitable initiating groups include mostly hydroxy moieties or other weak acids that are transferred to the initiator anion in the presence of a suitable base. In combination with alcohols, phosphazene bases are found to be highly efficient catalysts for ring-opening polymerization of cyclosiloxanes in organic solvents by proton abstraction and formation of a large and soft counterion [37,39,40,50]. Accordingly, the combination of (surface-bound) hydroxy groups with a phosphazene base seems to be a suitable approach for the development of a method to create end-functional PDMS chains and PDMS-based polymer brushes. The general reaction scheme and the respective chain polymerization mechanism is shown for the case of neopentyl alcohol in Scheme 1.

**Scheme 2:**
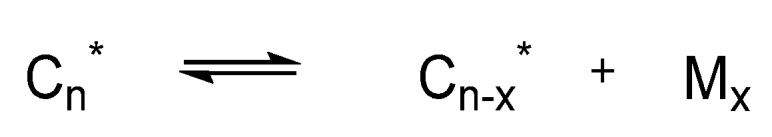
Ring–chain equilibrium in the anionic ring-opening polymerization of cyclosiloxanes. One monomeric unit M represents one -SiO(CH_3_)_2_- unit, while *n* and *x* give the numbers of repeating units in linear chains and ring molecules.

The mechanism of this reaction is well-elaborated [26,32,33,51]. The phosphazene base and the initiating alcohol form a phosphazenium alcoxide (initiating species I) which rapidly initiates the polymerization by nucleophilic attack at the silicon atom of a cyclic monomer M_4_ and formation into a phosphazenium silonate. This silanolate (active species) can open more monomer rings and thereby the active chain is extended. The reaction possesses several side reactions such as the formation of macrocycles. Because the propagation of the chains is reversible also backbiting of active centers with its own chain is possible. Thus, the reaction is a thermodynamically controlled ring–chain equilibration reaction between active linear polymer chains (Cn*) and ring species (Mx) with *x* monomeric units as described by the Jacobson-Stockmayer theory [33,50,52,53,54] and shown in Scheme 2. Numerous earlier works [21,26,33,53] have demonstrated that the anionic ROP of cyclosiloxanes can be seen as an excellent model system for this theory.

The concentration of active polymer chains can be assumed to be equal to the amount of added initiator, because alcohol and base are utilized in an equal amount. In general, the equilibrium constant $K_{x}$ for a cycle monomer with *x* repeating units $M_{x}$ is given by [21,33]

(2) $$K_{x}=\frac{\left[C_{n-x}{}^{*}\right]\cdot \left[M_{x}\right]}{\left[C_{n}{}^{*}\right]}\;\;\;$$

where $\left[M_{x}\right]$ is the concentration of cyclosiloxane rings with x repeating units and $\left[C_{n}{}^{*}\right]$ and $\left[C_{n-x}{}^{*}\right]$ are the concentration of active polymer chains with n and (n-x) repeating units, respectively.

For a Flory distribution of chain lengths in the linear part of the equilibrium, Equation (2) reduces to [21,33]

(3) $$K_{x}=\frac{\left[M_{x}\right]}{p^{x}}\;\;$$

with p as the extent of functional groups in the chain polymer, which for the present system can be described by the polymerization degree $X_{n}$ as [21,33]

(4) $$p=1-\frac{1}{X_{n}}\;$$

For practical reasons, in the following, the equilibrium constant is considered as effective equilibrium constant $K_{eff}$ for the formation of every x-meric ring with x being a small number [21]. Based on the ring chain equilibrium, the final mass-related yield *Φ* of the linear polymer chains is determined by

(5) $$\Phi =1-\frac{{\left[M_{x}\right]}_{\mathrm{eq}}\;\cdot \;M_{x}}{{\left[M_{4}\right]}_{0}\;\cdot \;M_{4}}.$$

(6) $$=1-\frac{K_{eff}\;\cdot \;p^{x_{eff}}}{{\left[M_{4}\right]}_{0}}\;\cdot \;\frac{M_{x}}{M_{4}}\;$$

with the initial monomer concentration ${\left[M_{4}\right]}_{0}$, the molar mass of the applied monomer $M_{4}$ and the average molar mass of rings in the equilibrium $\bar{M_{x}}$. Thus, the yield of the obtained linear polymer strongly depends on the initial monomer concentration. A simple way to experimentally confirm the relation in Equation (4) for M_4_ in toluene catalyzed by P_4_-*t*-Bu is to vary ${\left[M_{4}\right]}_{0}$ on the one hand and monomer to initiator ratio n_M_/n_I_ on the other hand.

With the yield and *M*_n_ obtained by SEC measurements the equilibrium polymer concentration [C]_eq_ and ring concentration [M_x_]_eq_ can be calculated according to Equations (7) and (8), respectively.

(7) $${\left[C\right]}_{\mathrm{eq}}=\frac{\Phi \;\cdot \;{\left[M_{4}\right]}_{0}\;\cdot \;M_{4}}{M_{n}}\;$$

(8) $${\left[M_{x}\right]}_{\mathrm{eq}}\approx {\left[M_{4}\right]}_{0}\;\cdot \;\left(1-\Phi \right)={\left[M_{4}\right]}_{0}-\frac{{\left[C\right]}_{\mathrm{eq}}\;\cdot \;M_{n}}{M_{4}}\;$$

In order to find suitable parameters for the surface-initiated ring-opening polymerization of M_4_; the ring–chain equilibrium as well as the possibility for molar mass control is investigated on a model solution-based polymerization. As a model initiator, 2,2-dimethyl-1-propanol (neopentyl alcohol, NeOH) is chosen to get access to the obtained molar mass *M*_n_ by end group analysis of ^1^H-NMR. Additionally, the molar mass distribution is determined by SEC relative to PS standards under application of the Mark–Houwink relationship.

#### 3.1.1. Ring–Chain Equilibrium

The influence of the initial monomer concentration on the ring–chain equilibrium of the solution-based model reaction is investigated to find a suitable concentration regime for the SI–ROP. Therefore, the ROP of M_4_ is investigated using P_4_-*t*-Bu as catalysts, NeOH as initiator and toluene as a solvent in different monomer concentrations. While silanols would principally be an apparently obvious alternative to serve as model initiators, they hold the general drawback to tendentially dimerize. In one set of experiments, the monomer-to-initiator ratio is kept constant at n_M_/n_I_ = 25 (Series 1). After the equilibrium is reached, the base is removed by quenching the reactive silanolate end group with chlorosilane. Low molecular oligomers and rings are separated from linear polymers and macrocycles by precipitation in cold MeOH.

**Figure 1:**
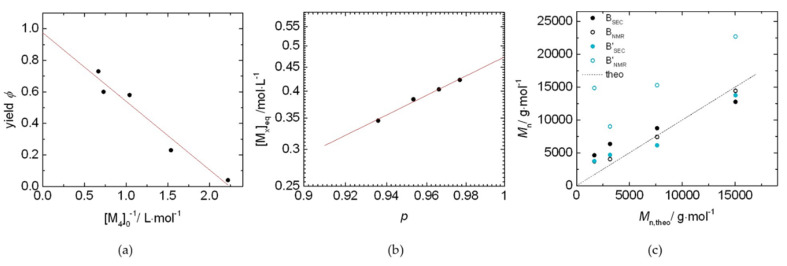
(**a**) Yield *Φ* as a function of the reciprocal monomer concentration for the ROP of M_4_ in toluene with NeOH as initiator and P_4_-*t*-Bu as catalyst for different monomer concentrations ([M_4_]_0_ and constant monomer-to-initiator ratio n*_M_*/n*_I_* = 25. (**b**) Double logarithmic plot of the ring concentration [M_x_]_eq_ over the extent of active groups p with linear fit function (red line) for the ROP of M_4_ in toluene ([M_4_]_0_ = 0.96 mol∙L^−1^), NeOH as initiator and P_4_-*t*-Bu as catalyst. (**c**) Molar mass *M*_n_ (obtained by SEC and ^1^H-NMR) as a function of *M*_n,theo_ for the ROP of M_4_ in toluene ([M_4_]_0_ = 0.96 mol∙L^−1^) with NeOH as initiator and two different catalysts (B: P_4_-*t*-Bu and B’: P_2_-*t*-Bu).

From Equation (5), the yield *Φ* is expected to decrease with a decreasing monomer concentration. In Figure 1a, the yield is plotted as a function of the reciprocal initial monomer concentration. With an increasing dilution, the ring–chain equilibrium shifts to the side of cycles, and the obtained yield decreases linearly until a critical monomer concentration [M_4_]_0_ is reached. Below this cut-off-concentration, virtually no polymer chains are formed. From the intercept at *Φ* = 0, the cut-off concentration can be determined to be [M_4_]^#^ = 0.45 mol∙L^−1^. This information is important for the identification of the suitable concentration regime for the SI–ROP. From the slope of the graph, we learn that $\frac{d\Phi }{d{\left[M_{4}\right]}_{0}^{-1}}={\left[M_{x}\right]}_{\mathrm{eq}}$∙$\frac{M_{x}}{M_{4}}$ = 0.43 mol∙L^−1^ which is close to the result for [M_4_]^#^. From Equation (3), it follows that ${\left[M_{x}\right]}_{\mathrm{eq}}\;\approx {\left[M_{4}\right]}^{\#}$ for not too short polymers (*p* → 1) and *x* being a small number. Thus, $\frac{M_{x}}{M_{4}}$ is close to 1 and mostly small rings with four to six repeating units are formed.

#### 3.1.2. Molar Mass Control

The ring–chain equilibrium is known to hamper the control of molar mass [26,31]. The control of molar mass is investigated by performing the ROP of M_4_ with P_4_-*t*-Bu (B) as catalysts and NeOH as initiator at constant monomer concentration ([M_4_]_0_ = 0.96 mol∙L^−1^) for different *n*_M_*/n*_I_ ratios (Series 2). The molar mass of the polymer after precipitation is determined using end group analysis of ^1^H-NMR and SEC (see Figures S1–S4). The obtained *M*_n_,_NMR_ and *M*_n_,_SEC_ as functions of the theoretically determined molar mass *M*_n_,_theo_ are shown in Figure 1c. The yields in the expected regime for the chosen monomer concentration and *M*_n_ obtained by ^1^H-NMR are in good agreement with *M*_n_ obtained by SEC. In general, for the chosen n*_M_*/n*_I_* regime, the final molar mass is close to the value of *M*_theo_ which is calculated based on the employed n*_M_*/n*_I_* ratio. Only for very small n*_M_*/n*_I_* ratios is the molar mass control is less optimal, because low molar mass polymers are separated from the product during the purification step as they do not precipitate into cold MeOH.

With the molar masses and Equation (8), the equilibrium ring concentration [M_x_]_eq_ is determined for each n*_M_*/n*_I_* ratio. According to Equation (3), an effective number of monomeric units $x_{eff}$ can be obtained from the slope of the linear fit from the plot of log([M_x_]_eq_) as a function of log(p) as shown in Figure 1b. For the present system, a value of $x_{\mathrm{eff}}$ = 4.63 is obtained. Thus, mostly rings with four and five siloxane units are formed in the ROP of M_4_ as already assumed in Section 3.1.2. Moreover, from the intercept, a value for $K_{eff}$ = 0.47 mol∙L^−1^ is obtained, which is thus similar to the cut-off concentration [*M*_0_]^#^ as determined from the results from experimental Series 1 and as again would be expected from Equation (2) with *p* ~ 1 and *x* as a small number.

Since the polymerization rate for the ROP of cyclosiloxanes increases with the size of the counterion [21,26], the performance of two different phosphazene bases, P_4_-*t*-Bu (ionic radius = 4.7 Å [55], p*K*_BH_+ = 41.9 [56]) and P_2_-*t*-Bu (ionic radius = 3.3 Å [55], p*K*_BH_+ = 33.5 [56]), is investigated. The ROP of M_4_ is performed in toluene at constant monomer concentration (([M_4_]_0_ = 0.96 mol∙L^−1^) with P_4_-*t*-Bu and P_2_-*t*-Bu (B’), respectively, and varying n*_M_/*n*_I_*. Figure 1c shows the molar masses as a function of *M*_n,theo_. The yield does not show a significant change with respect to the initial n*_M_*/n*_I_* ratio for both catalysts and is in the expected regime according to the chosen monomer concentration. However, the catalysts differ in the resulting molar mass. Whereas for P_4_-*t*-Bu, *M*_n,NMR_ is in good agreement with *M*_n,SEC_, *M*_n,NMR_ is constantly higher than *M*_n,SEC_. By using the model of end group analysis, we compare the signal for the CH_3_-group of the initiator NeOH with the signal of the Si-CH_3_ group assuming that every polymer chain possesses the same initiating group. The high *M*_n,NMR_ indicates the presence of macrocycles or another initiator, possibly due to water residues in the P_2_-*t*-Bu solution, as water in combination with strong bases is known to initiate the ROP of cyclosiloxane [36,38]. Consequently, the study focuses on the employment of P_4_-*t*-Bu as a catalyst in the following.

### 3.2. Surface-Initiated Ring-Opening Polymerization

**Scheme 3:**
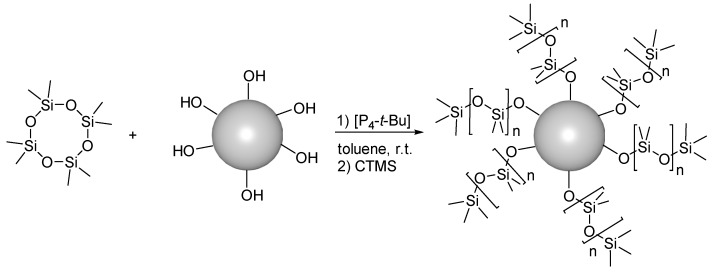
Base catalyzed surface-initiated (SI)–ROP of M_4_ initiated by SiO_2_ surface of NP.

The knowledge of the anionic ROP catalyzed by phosphazene bases is used to develop a novel route for the surface functionalization of nanoparticles with PDMS brushes. The polymerization is initiated by functional groups at the surface of the nanoparticles as presented in Scheme 3.

Instead of alcohols, surface hydroxy groups are used as initiators. The SI–ROP is performed on SiO_2_ and SiO_2_ coated spindle-like Fe_2_O_3_ particles, because the silica surface naturally contains hydroxy groups.

**Figure 2:**
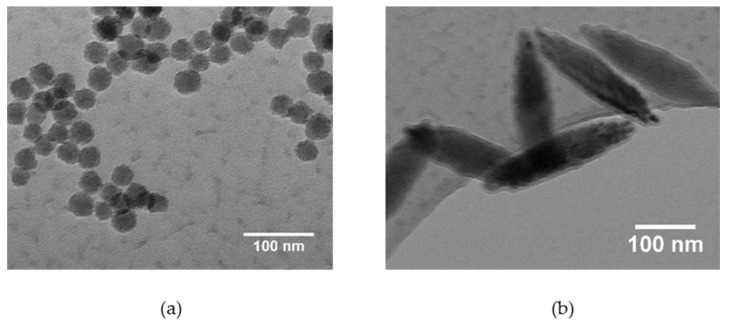
TEM image of (**a**) SiO_2_ particles and (**b**) SiO_2_@α-Fe_2_O_3_ particles.

SiO_2_ particles are synthesized by decomposition of TEOS using a modified Stöber process [46]. Core-shell particles SiO_2_@Fe_2_O_3_ are prepared using spindle-like α-Fe_2_O_3_ particles synthesized by the hydrothermal composition of iron (III) chloride using the method developed by Ozaki et al. [47]. After citric acid stabilization hematite spindles are covered with a thin silica shell based on the route of Philipse [49] and Wagner [48]. Figure 2a,b shows TEM images of the obtained SiO_2_ and SiO_2_@Fe2O_3_ particles after synthesis. The SiO_2_ spheres show a diameter of *d* = 26.17 ± 3.5 nm. The spindle-like hematite core possesses a length of *L* = 268.2 ± 19.3 nm and a width of *a* = 67.4 ± 8.1 nm. The thickness of the silica shell is observed to be *s* = 8.8 ± 1.6 nm. The purity of the SiO_2_ nanoparticles is checked by elemental analysis (see Table S1). Within the experimental error, the values for nitrogen and carbon content give no evidence for any impurities. ^1^H-NMR spectroscopy of silica particles (Figure S6) also only shows the characteristic signals of the solvent.

For the SI–ROP, SiO_2_ particles dispersed in toluene are initially mixed with the catalysts P_4_-*t*-Bu. The reaction is started by rapid addition of the monomer. After stirring the reaction mixture for an appropriate time (generally 24 h) at ambient temperature, the chains are deactivated by quenching with CTMS. The particles are collected via centrifugation and washed until no PDMS is detected in the supernatant. The supernatant is collected, reduced and precipitated into cold MeOH to analyze any free PDMS species.

**Figure 3:**
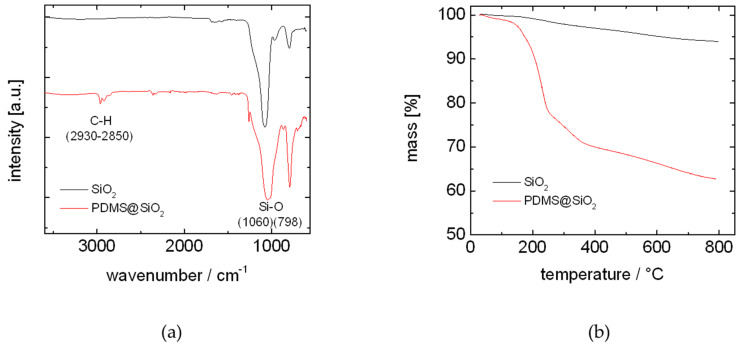
(**a**) ATR–IR spectrum and (**b**) TGA of pure silica particles and PDMS@SiO_2_ exemplary shown for the SI–ROP of M_4_ with [M_4_]_0_ = 1.49 mol∙L^−1^ and [B] = 1.49 × 10^−2^ mol∙L^−1^.

The presence of PDMS at the particle’s surface is qualitatively confirmed by ATR–IR spectroscopy. Compared to the particles prior the polymerization, PDMS@SiO_2_ show an additional characteristic C–H stretching vibration at 2930–2850 cm^−1^ which corresponds to the CH_3_ groups of the PDMS shown exemplary in Figure 3a for pure SiO_2_ particles and PDMS@SiO_2_ after the SI–ROP of M_4_ with [M_4_]_0_ = 1.49 mol∙L^−1^ and [B] = 1.49 × 10^−2^ mol∙L^−1^. Quantitative information is obtained from TGA under inert atmosphere by comparing the mass loss of pure SiO_2_, pure PDMS and PDMS@SiO_2_ (Figure 3b). Pure SiO_2_ is known to show a small characteristic mass loss due to the dihydroxylation of silanol groups [57]. For carefully isolated, washed and dried PDMS@SiO_2_ hybrid particles, the relative mass loss is drastically increased corresponding to an increase in the organic content in the sample due to the PDMS brush. In contrast, in blind experiments using linear preformed PDMS and SiO_2_ particles, we confirmed that pure physisorption of the polymer to the particle surface does not lead to such an increase.

With the relative mass loss of the PDMS@SiO_2_, Δ*m*, and of pure SiO_2,_ Δ*m*_SiO2_, respectively, and under proper consideration of the thermostable (inorganic) residue observed in pure PDMS under the same conditions (*µ*_PDMS_ = 0.8), the PDMS content on the particle *µ*_PDMS,p_ can be determined using Equation (9)

(9) $$\mu_{\mathrm{PDMS},p}=\frac{∆m-∆m_{{\mathrm{SiO}}_{2}}}{\mu_{\mathrm{PDMS}}}\;,$$

Accordingly, the yield of surface-attached PDMS, *Φ*_p_, can be calculated by using Equation (10)

(10) $$\Phi_{p}=\frac{m_{\mathrm{SiO}2}}{m_{M4,0}}\;\cdot \;\frac{1}{1-\mu_{\mathrm{PDMS},p}}\;,$$

where $m_{{\mathrm{SiO}}_{2}}$ = mass of SiO_2_ and $m_{M_{4,0}}$ = mass of employed monomer.

As an inherent property of the underlying polymerization mechanism, the SI–ROP of most cyclosiloxanes is accompanied by a series of side reactions that are difficult or impossible to circumvent, the most relevant of these is the cyclic backbiting reaction investigated in Section 3.1. As a result, if parasitic initiation in solution is possible, the formation of free polymer that is not attached to the particle surface is possible. Under these conditions, the SI–ROP is not surface-confined.

In the experiments reported here, the formation of free PDMS in solution is confirmed in all cases where [M_4_]_0_ > [M_4_]^#^. It is expected that the corresponding parasitic initiation either occurs from impurities such as water or siloxanates, or from the monomers themselves under activation by the P_4_ catalyst, resulting in the formation of macrocycles. In order to obtain more information, the free PDMS component is isolated by precipitation of the reaction solution (after particle separation by centrifugation) in cold MeOH and analyzed via SEC.

The reaction is investigated regarding two different parameters. On the one hand, the influence of the monomer concentration on the SI–ROP reaction is investigated by performing the reaction with constant particle amount (initiator), base amount and M_4_ amount, thus constant monomer-to-initiator ratio, but with varying solvent amount (Series 3 and 4, respectively). On the other hand, the SI–ROP of M_4_ is further investigated regarding the monomer-to-initiator ratio. Therefore, the amounts of particles, base and solvent are kept constant and only the amount of M_4_ is varied (Series 5 and 6, respectively). Analogously, the SI–ROP is performed using spherical SiO_2_ particles as spindle-shaped SiO_2_@Fe_2_O_3_ particles in order to demonstrate the versatility of the approach. Compared to the SiO_2_ particles used in this study, the employed spindle-like SiO_2_@Fe_2_O_3_ particles are significantly larger and thus have a lower surface-to-volume ratio. Since the particle surface determines the number of initiator groups, the amount of M_4_ is adapted in order to get the same monomer-to-surface ratio as in the SI–ROP with SiO_2_ particles.

The surface-attached PDMS fraction *µ*_PDMS,p_ determined by TGA for the different monomer concentrations and ratios of monomer-to-particle concentration *υ*_P_ are listed in Table 1. For every monomer concentration in Series 3, PDMS is generated at the surface of particles with *µ*_PDMS,p_ between 37% and 42% for SiO_2_ and in Series 4 between 7% and 14% for SiO_2_@Fe_2_O_3_, respectively. Within the experimental error, a significant trend cannot be observed. For Series 5 and 6, both particle types also indicate an increase in surface-attached polymer fraction with increasing monomer concentration. The results indicate the presence of an equilibrium situation for PDMS at the particle surface which limits *µ*_PDMS,p_ with increasing monomer amount. The surface-attached PDMS contents are in total smaller for SiO_2_@α-Fe_2_O_3_ than for SiO_2_ but with regard to the surface area *A*_p_, the amount of PDMS per nm² for SiO_2_@α-Fe_2_O_3_ (4 × 10^−18^–9 × 10^−18^ mg∙nm²) is slightly higher than for SiO_2_ (1 × 10^−18^–4 × 10^−18^ mg∙nm²).

**Figure 4:**
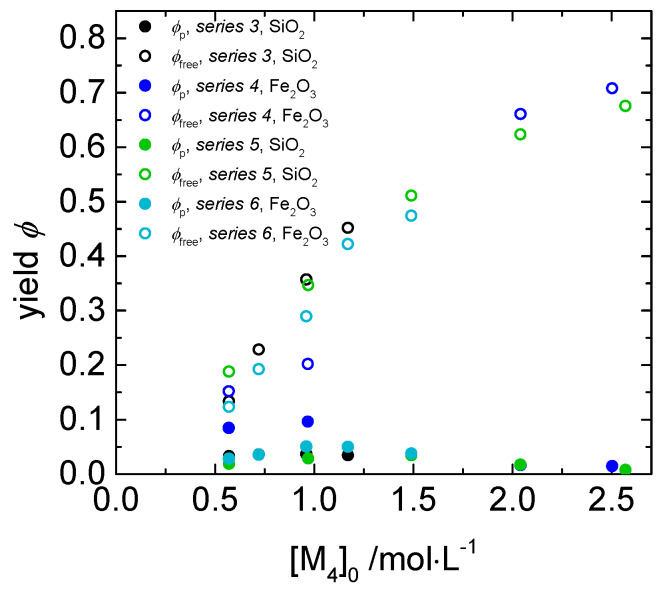
Yields of surface-attached PDMS and free PDMS as a function of monomer concentration for the SI–ROP of M_4_ on SiO_2_ and on SiO_2_@α-Fe_2_O_3_ obtained in Series 3 to 6.

Figure 4 compares the yield regarding PDMS at the particle and free PDMS in the solution for both particle systems. Compared to the yield of free PDMS *Φ*_free_, the yield of surface-attached PDMS *Φ*_P_ is with ~3.5% quite low and does not show a significant trend with [M_4_]_0_. In contrast, the yield of free PDMS increases with increasing monomer concentration [M_4_]_0_ due to the ring–chain equilibrium as already observed in the model solution-based polymerization in Section 3.1. For the yields for the surface-attached and free PDMS of SiO_2_@Fe_2_O_3_, the same trends as for the silica-based system are observed. While the yield of particle-bound PDMS is almost constant, the amount of free PDMS increases with increasing monomer concentration, as observed in Section 3.1.

**Figure 5:**
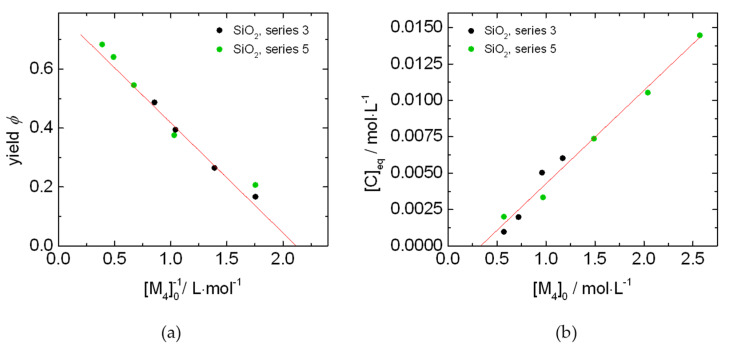
(**a**) Yield *Φ* as a function of the reciprocal monomer concentration and (**b**) polymer concentration from SI–ROP of M_4_ with SiO_2_ particles as a function of ${\left[M_{4}\right]}_{0}$ for Series 3 and 5.

By plotting *Φ*_f_ and *Φ*_P_ as a function of reciprocal [M_4_]_0_ (Figure 5a) it is observed, the values for Series 3 and 5, respectively, and of Series 4 and 6 (Figure S5a) form a master curve. With the linear regression of the master curve and Equation (4), a cut-off concentration [M_4_]_0,c_ of 0.46 mol∙L^−1^ for SiO_2_ and 0.47 mol∙L^−1^ for SiO_2_@Fe2O_3_ (see ESI, Figure S5) is determined, which is close to the ROP in the absence of particles. To get more information about the origin of the free PDMS, ^1^H-NMR spectroscopy is performed (Figure S9), but the spectra give no evidence for initiating impurities. Furthermore, the molar mass is measured and listed in Table 1 for the variation of solvent amount (Series 3 and 4) and for the variation of the monomer-to-initiator ratio (Series 5 and 6). The corresponding SEC curves are shown in the ESI (see Figures S7 and S8). With the yield and the molar mass *M*_n_,_free_, the molar polymer concentration [C]_eq_ can be determined using Equation(7). In Figure 5b the results for [C]_eq_ are plotted over [M_4_]_0_. With increasing [M_4_]_0_ the polymer concentration [C]_eq_ increases linearly, while a similar correlation is not found for the concentration of any of the other reaction partners, such as the particles, solvent, or catalyst. In addition, the water content of the reaction mixtures was in the low μmol range in all cases, and thus well below the concentration of free polymer chains. Further, elemental analysis concerning elements C, N and H, performed on our SiO_2_ particles confirms the absence of organic impurities. A plausible explanation for these observations and the linear correlation between equilibrium polymer concentration and initial monomer concentration is a polymerization mechanism based on activated monomer initiation that leads to the formation of macrocycles by repeated monomer insertion. In this case, the inverse slope of the graph, [M_4_]_0_/d[C]_eq_, gives a value for the degree of polymerization. Using this method, we calculate an average molar mass of 46000 g∙mol^−1^ for SiO_2_ and 43000 g∙mol^−1^ SiO_2_@α-Fe_2_O_3_ for the free polymer chains.

**Figure 6:**
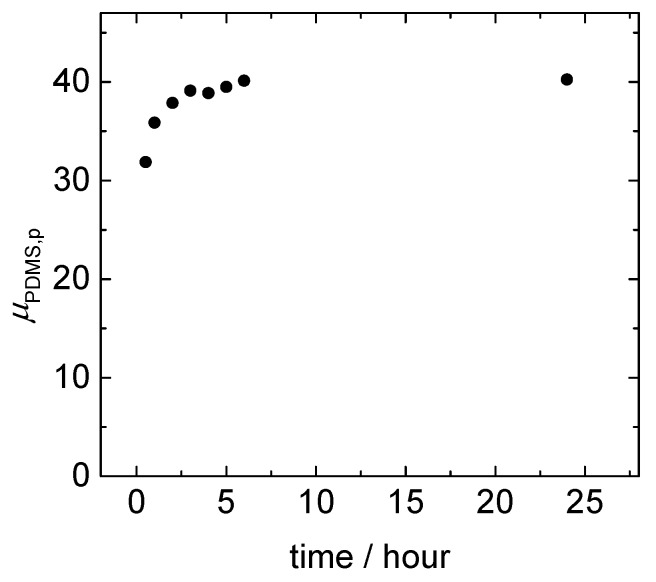
Surface-attached polymer fraction *µ*_PDMS,p_ as a function of reaction time (Series 7).

In order to learn more about the SI–ROP of M_4_ using SiO_2_ particles, the reaction is investigated over time (Series 7) by determining the relative polymer fraction *µ*_PDMS,p_ after consecutive different periods of reaction time. The results are illustrated in Figure 6. Most of the surface-attached PDMS is generated in the first hour. During this time, *µ*_PDMS,p_ increases to 36%. Afterwards, the polymer content slowly increases with a maximum *µ*_PDMS,p_ of 40% after 6 h. Compared to the particle-free polymerization, the formation of surface-attached chains is considerably slower.

## 4. Discussion

In this work, the anionic SI–ROP of M_4_ is used to functionalize nanoparticles with PDMS polymer brushes. Therefore, surface hydroxy groups are used as initiators and a strong phosphazene base is utilized as catalyst. Caused by difficulties arising from the particular polymerization-depolymerization thermodynamics of the anionic ring-opening polymerization of most cyclosiloxanes, the conditions that enable the creation of a well-defined, surface-attached polymeric brush based on a grafting-from mechanism need a careful choice, and accordingly, the SI–ROP of polysiloxanes has not been successfully reported before.

For this purpose, we deeply explore the conditions based on a solution-based model polymerization using neopentyl alcohol (NeOH) as initiator. The choice of initiator is motivated to enable the calculation of the NMR-equivalent number average molar mass, in order to compare it to the corresponding result obtained by universal calibration SEC. In our results, the mass-related yield of PDMS increases steadily with increasing [M_4_]_0_ (Figure 1), which is in accordance with the expectations as outlined in Section 3.1. No polymer formation is observed as long as the monomer concentration is below the cut-off concentration [M_4_]^#^ that is determined to be at around 0.45 mol∙L^-1^ under the conditions of choice. The higher the monomer concentration, the more the polymer formation is favored.

The possibility to control the molar mass of the final polymer fraction by choice of the initial molar monomer-to-initiator ratio is tested for two different, structurally and chemically closely related bases, P_4_-*t*-Bu and P_2_-*t*-Bu. Whereas, for P_4_-*t*-Bu, the results for *M*_n_,_NMR_ and *M*_n_,_SEC_ are throughout both in good agreement with the theoretically expected molar mass, for P_2_-*t*-Bu the value obtained for *M*_n_,_NMR_ is significantly higher than *M*_n_,_SEC_, indicating that not every polymer chain possess the neopentyl alcoholate as the end group. This is an indication for unwanted side reactions that are almost absent as found from the results on polymers catalyzed with P_4_-*t*-Bu, and the latter is consequently employed in the experiments of the following up experiments.

With the knowledge gained, a method for the PDMS functionalization of nanoparticles by using surface hydroxy groups as initiators for the ROP of M_4_ is developed and demonstrated using spherical SiO_2_ particles and silica-coated Fe_2_O_3_ spindles as base. The surface-attached PDMS content *µ*_PDMS,p_ is quantified by TGA measurements. The SI–ROP is successfully applied for both particle types, but also the formation of free PDMS by some kind of parasitic solution initiation is observed in each system. The SI–ROP is investigated regarding the variation of solvent amount (Series 3 and 4) and the ratio between monomer and particles (Series 5 and 6). For both particle systems, the obtained *µ*_PDMS,p_ differs not substantially between the entries.

We carefully considered the possibility of polymer initiation occurring from hydroxy-functional impurities, such as water or ethanol, in each of the reaction components, including particles, monomer, solvent and catalyst. The purity of particles was confirmed by elemental analysis. Particularly the water content of the particle dispersion was repeatedly shown to be as low as (85 ± 2) ppm with a Karl-Fischer titrator. In addition, the only correlation between the final polymer concentration and the fraction of a given compound is found for the monomer concentration, and the yield of isolated free PDMS increases with increasing monomer concentration, in a similar way as observed for the model solution-based polymerization. In the absence of an external solution-based initiator, it is anticipated that the formation of macrocycles by a self-initiation / insertion mechanism might be the cause. The linear correlation of the polymer concentration [C]_eq_ and the monomer concentration [M_4_]_0_ strengthens this hypothesis. It is further confirmed by additional experiments performed by the SEC with simultaneous refractive index (RI) and multi-angle static light scattering (MALLS) detection. By direct comparison of the mass-average molar mass obtained from the (absolute) MALLS signal to the result of the (relative) result related to the RI signal after Mark–Houwink correction, the absolute value is systematically higher, and we observe a slope of 1.40 in this correlation. The ratio between the hydrodynamic radius of a macrocycle in relation to its linear analog is expected to be reduced by a factor of $\sqrt{2}$.

In all investigated series, the cut-off monomer concentration [M_4_]^#^ and the equilibrium concentration of small rings [M_x_]_eq_ happen to be of constant value similar to the observation in solution and apparently hardly influenced by the presence of the particles, indicating the presence of a limiting factor for the surface-attached fraction of PDMS under these reaction conditions.

To get additional information on the mechanism, the SI–ROP is investigated kinetically by measuring the PDMS content as a function of reaction time. Compared to the appearance of the free polymer in the solution that quickly reaches a near-equilibrium concentration within a few minutes, the fraction of the surface-attached polymer *µ*_PDMS,p_ increases significantly slower, with the main growth period during the first hour of the reaction time.

The results indicate that the mechanism behind the generation of surface-grafted PDMS brushes on SiO_2_-coated surface of nanoparticles is not only influenced by the fast ROP kinetics of cyclosiloxanes and the ring–chain equilibrium of the particles, but in addition by a concurring reaction initiated by the OH-groups of the particle surface; while, from a fundamental acid-base consideration, the initiation from a Si-OH group as found on the particle surface is principally possible, the missing flexibility of the corresponding geometry and the confinement on the particle surface give rise to a limited effectivity of surface initiation.

**Scheme 4:**
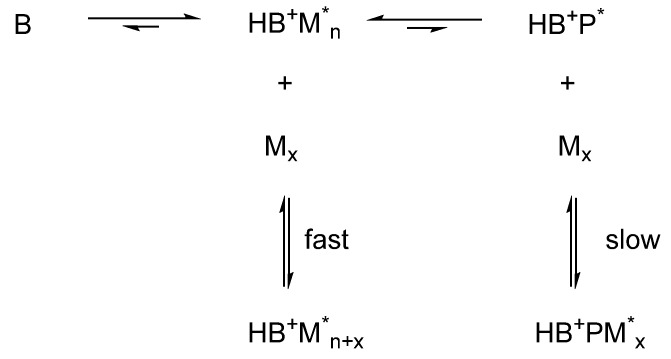
Proposed equilibria in the SI–ROP of M_4_.

The presence of free PDMS and surface-attached PDMS indicates the development of various equilibria in the reaction as illustrated in Scheme 4. The catalysts P_4_-*t*-Bu base (B) is in equilibrium with its protonated species, which can be formed with the free PDMS/initiator (HB^+^M^*^_n_) species or with the hydroxy group at the particle surface (HB^+^P^*^). Because “free” species are more easily accessible than particle surface groups, the base favor to form a free HB^+^M^*^_n_. Moreover, each active species can open further rings and form a long linear polymer chain which is known to be a ring–chain equilibrium. Based on the kinetic measurement, this equilibrium is faster for the free species than for the PDMS at the particle surface. Due to the equilibrium between HB^+^M^*^_n_ and HB^+^P^*^ the ratio of monomers which is converted onto the particle surface is limited. An increase in the applied monomer amount only leads to an increase in the formation of free PDMS.

If the proposed mechanism is feasible, it can be anticipated that with an increasing portion of surface-attached Si-O-groups that now are equipped with one or more M_4_ units, the surface-attached oligomers will more and more contribute to the faster equilibrium. Accordingly, an increase of the polymer fraction might generally be possible by quickly adding an additional portion of the monomer to the reaction mixture after reaching the equilibrium of free polymer, surface-bound polymer and small rings.

**Figure 7:**
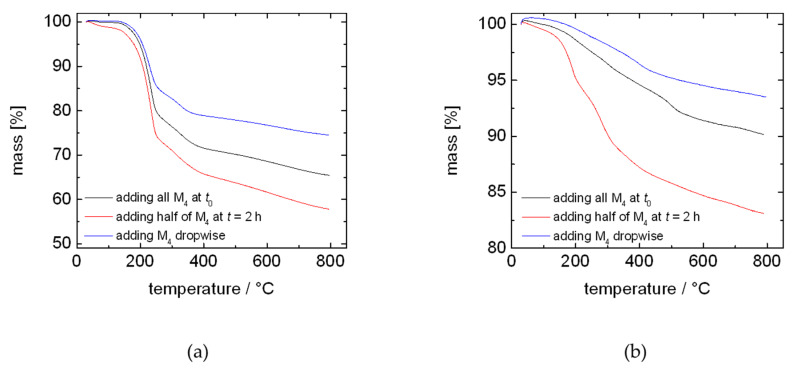
TGA for (**a**) PDMS@SiO_2_ and (**b**) PDMS@SiO_2_@Fe_2_O_3_ synthesized with different injection methods.

In order to investigate this, the mode of monomer injection, that in all previously described experiments occurs by rapid injection at once, is varied. In direct comparison to the usual injection in one portion, the reaction is performed on the one hand by adding the monomer M_4_ dropwise over a period of 30 min under stirring, and on the other hand by injecting the monomer M_4_ in two portions, the first one at the reaction start, and the second portion after two hours. The TGA thermograms for these samples that are prepared with the same total monomer amount are compared in Figure 7a,b. While the dropwise addition of M_4_ leads to a decrease in the final PDMS content for both particle types, the addition of a second fraction of the monomer after system equilibration results in an increase of polymer fraction. For the hematite particle, this increase means an almost double mass fraction of PDMS.

All observations are in correspondence for a general cut-off concentration of about 0.45 mol∙L^−1^ for M_4_ in toluene at 25 °C. Below this cut-off concentration, the ring–chain equilibrium is far on the ring side. By adding the monomer dropwise, virtually no long-chain (or macrocycle) PDMS is generated until reaching this concentration. Upon further slow monomer addition, a preferably free polymer is formed. In contrast, by adding the monomer in two portions with a delay of two hours, when PDMS chains are already generated at the particle surface. Assuming that the surface initiation is the rate-limiting step for the brush formation, now the surface reaction is more competitive.

## 5. Conclusions

In this paper, a method for the surface functionalization of nanoparticles with PDMS by anionic SI–ROP of M_4_, catalyzed by a phosphazene base, is developed. Suitable reaction parameters are investigated by using a model solution-based polymerization consisting of M_4_, NeOH as initiator and under comparison of two different base catalysts. The findings are applied to identify useful reaction conditions for a successful surface-initiated ROP of M_4_ using hydroxy groups at the surface of SiO_2_ particles and silica-coated Fe_2_O_3_ particles, for the generation of PDMS brushes. The SI–ROP is shown to be an equilibrium reaction between small rings, free polymer chains/macrocycles and surface-attached PDMS, which leads to a limit for the PDMS content of the particles. Under these considerations, the PDMS content can be further increased by the delayed addition of more monomers. The results for the equilibrium polymer fraction for the different particle types are in accordance with a similar surface coverage.

The SI–ROP of M_4_ developed in this work is a promising method for the surface functionalization of different kinds of nanoparticles with cyclosiloxanes of different polarity or functionality, as we can expect it to be transferable to other particle core materials and shapes and to other monomers that can be polymerized by an analogous mechanism. By using functionalized cyclosiloxanes, the introduction of various functional groups is possible.

## Figures and Tables

**Table 1:** Polydimethylsiloxane (PDMS) content *µ*_PDMS,p_ molar mass of free polymer *M*_n,free_ and PDI for the SI–ROP of M_4_ for the variation of solvent (Series 3 and 4) and the variation of monomer amount (Series 5 and 6) using SiO_2_ and SiO_2_@α-Fe_2_O_3_.

**SiO_2_**											
	**Series 3**					**Series 5**					
${\left[M_{4}\right]}_{0}$ **mol∙L^−1^**	${\left[M_{4}\right]}_{0}$ **/υ_p_ mmol∙mg^−1^**	$\mu_{\mathrm{PDMS},p}$	***m*_PDMS_/*A*_p_ mg∙m^−2^**	***M*_n,free_ g∙mol^−1^**	**PDI**	${\left[M_{4}\right]}_{0}$ **mol∙L^−1^**	${\left[M_{4}\right]}_{0}$ **/υ_p_ mmol∙mg^−1^**	$\mu_{PDMS,p}$	***m*_PDMS_/A_p_ mg∙m^−2^**	***M*_n,free_ g∙mol^−1^**	**PDI**
0.57	0.045	0.371	4.44	23252	1.72	0.57	0.011	0.069	0.96	15802	2.09
0.72	0.045	0.395	4.65	24586	1.85	0.97	0.023	0.129	1.42	29864	2.04
0.96	0.045	0.413	4.57	20174	1.68	1.49	0.045	0.402	4.44	30593	1.97
1.17	0.024	0.421	4.36	25967	2.02	2.04	0.090	0.403	4.45	35748	2.15
1.49	0.024	0.402	4.08	30593	1.97	2.57	0.180	0.37	4.09	35521	2.04
**SiO_2_@Fe_2_O_3_**											
		**Series 4**				**Series 6**					
${\left[M_{4}\right]}_{0}$ **mol∙L^−1^**	${\left[M_{4}\right]}_{0}$ **/υ_p_ mmol∙mg^−1^**	$\mu_{PDMS,p}$	***m*_PDMS_/*A*_p_ mg∙m^−2^**	***M*_n,free_ g∙mol^−1^**	**PDI**	${\left[M_{4}\right]}_{0}$ **mol∙L^−1^**	${\left[M_{4}\right]}_{0}$ **/υ_p_ mmol∙mg^−1^**	$\mu_{PDMS,p}$	***m*_PDMS_/*A*_p_ mg∙m^−2^**	***M*_n,free_ g∙mol^−1^**	**PDI**
0.57	0.013	0.056	3.73	20493	2.74	0.57	0.003	0.073	4.73	19420	1.67
0.72	0.013	0.083	5.54	25428	1.67	0.97	0.006	0.130	8.64	23907	1.73
0.96	0.013	0.138	9.12	21355	2.31	1.49	0.013	0.091	4.57	35289	1.87
1.17	0.013	0.139	9.21	26830	1.71	2.04	0.026	0.083	5.53	37084	1.79
1.49	0.013	0.091	6.05	35289	1.87	2.57	0.052	0.082	3.73	34168	1.84

## Supplementary Materials

The following are available online at https://www.mdpi.com/2073-4360/12/4/787/s1, Figure S1: ^1^H-NMR spectra of ROP of M_4_ in toluene with NeOH as initiator, P_4_-*t*-Bu as catalyst measured in CDCl_3_ for an monomer-to-initiator ratio of a) n*_M_/*n*_I_* = 5, b) n*_M_/*n*_I_* = 10, c) n*_M_/*n*_I_* = 25 and d) n*_M_/*n*_I_* = 50, Figure S2: Molar mass distribution of ROP of M_4_ in toluene with NeOH as initiator, P_4_-*t*-Bu as catalyst and different monomer-to-initiator ratios measured by SEC, Figure S3: ^1^H-NMR spectrum of ROP of M_4_ in toluene with NeOH as initiator, P_2_-*t*-Bu as catalyst measured in CDCl_3_ for an monomer-to-initiator ratio of a) n*_M_/*n*_I_* = 5, b) n*_M_/*n*_I_* = 10, c) n*_M_/*n*_I_* = 25 and d) n*_M_/*n*_I_* = 50, Figure S4: Molar mass distribution of ROP of M_4_ in toluene with NeOH as initiator, P_2_-*t*-Bu as catalyst and different monomer-to-initiator ratios measured by SEC, Figure S5: a) Yield as a function of the reciprocal monomer concentration and b) polymer concentration from SI–ROP of M_4_ with SiO_2_@Fe_2_O_3_ particles as a function of [M_4_]_0_ for *series 4* and *series 6*, Figure S6: ^1^H-NMR spectrum of SiO_2_ particles measured in CDCl_3_, Figure S7: Molar mass distribution of free Polymer obtained during SI–ROP of M_4_ with SiO_2_ particles as a function of ${\left[M_{4}\right]}_{0}$ for a) *series 3* and b) *series 5*, Figure S8: Molar mass distribution of free PDMS obtained during the SI–ROP of M_4_ with SiO_2_@Fe_2_O_3_ particles as a function of ${\left[M_{4}\right]}_{0}$ for a) *series 4* and b) *series 6*, Figure S9: Example for ^1^H-NMR spectrum of free PDMS obtained during the SI–ROP of M_4_ with SiO_2_ particles measured in CDCl_3_, Table S1: Results of elemental analysis for SiO_2_ and SiO_2_@α-Fe_2_O_3_, Table S2: Yield of surface-attached PDMS and free PDMS for the SI–ROP of M_4_ performed with different injection methods.

## References

[1] Mefford O.T., Vadala M.L., Goff J.D., Carroll M.R.J., Mejia-ariza R., Caba B.L., Pierre T.G.S., Woodward R.C., Davis R.M., Riffle J.S. (2008). Stability of Polydimethylsiloxane-Magnetite Nanoparticle Dispersions Against Flocculation: Interparticle Interactions of Polydisperse Materials. Langmuir.

[2] Möller M., Nederberg F., Lim L.S., Kånge R., Hawker C.J., Mo M., Hedrick J.L., Gu Y., Shah R., Abbott N.L. (2001). Stannous (II) Trifluoromethane Sulfonate: A Versatile Catalyst for the Controlled Ring-Opening Polymerization of Lactides: Formation of Stereoregular Surfaces from Polylactide “Brushes”. J. Polym. Sci. Part A Polym. Chem..

[3] Alexander S. (1977). Adsorption of chain polymers with a polar head: A scaling description. J. Phys..

[4] De Gennes P.G.G. (1980). Conformations of Polymers Attached to Interface. Macromolecules.

[5] Thünemann A.F., Schütt D., Kaufner L., Pison U., Möhwald H. (2006). Maghemite Nanoparticles Protectively Coated with Poly(ethylene imine) and Poly(ethylene oxide)-bloc-poly(glutamic acid). Langmuir.

[6] Si S., Kotal A., Mandal T.K., Giri S., Nakamura H., Kohara T. (2004). Size-Controlled Synthesis of Magnetite Nanoparticles in the Presence of Polyelectrolytes. Chem. Mater..

[7] Mcewan M., Green D. (2009). Rheological impacts of particle softness on wetted polymer-grafted silica nanoparticles in polymer melts. Soft Matter.

[8] Yoon K.R., Chi S., Lee K., Lee J.K., Kim J., Koh Y., Joo S., Yun S., Choi I.S. (2003). Surface-initiated ring-opening polymerization of p-dioxane from gold and silicon oxide surfaces. J. Mater. Chem..

[9] Dai Q., Lam M., Swanson S., Yu R.H.R., Milliron D.J., Topuria T., Jubert P.O., Nelson A. (2010). Monodisperse cobalt ferrite nanomagnets with uniform silica coatings. Langmuir.

[10] Schmidt A.M. (2005). The synthesis of magnetic core-shell nanoparticles by surface-initiated ring-opening polymerization of ε-caprolactone. Macromol. Rapid Commun..

[11] Green D.L., Mewis J., Engineering C., Uni V., Way E., Charlottes V. (2006). Connecting the Wetting and Rheological Behaviors of Poly(dimethylsiloxane)-Grafted Silica Spheres in Poly(dimethylsiloxane) Melts. Langmuir.

[12] Cho Y.K., Park E.J., Kim Y.D. (2014). Removal of oil by gelation using hydrophobic silica nanoparticles. J. Ind. Eng. Chem..

[13] Mouhli A., Ayeb H., Othman T., Fresnais J., Dupuis V., Nemitz I.R., Pendery J.S., Rosenblatt C., Sandre O., Lacaze E. (2017). Influence of a dispersion of magnetic and nonmagnetic nanoparticles on the magnetic Fredericksz transition of the liquid crystal 5CB. Phys. Rev. E.

[14] Brillouin L.L., Saclay C.E.N., Sur G., Cedex Y. (1991). Building of Grafted Layer. 1. Role of the Concentration of Free Polymers in the Reaction Bath. Macromolecules.

[15] Prucker O., Rühe J. (1998). Mechanism of Radical Chain Polymerizations Initiated by Azo Compounds Covalently Bound to the Surface of Spherical Particles. Macromolecules.

[16] Shah R.R., Merreceyes D., Husemann M., Rees I., Abbott N.L., Hawker C.J., Hedrick J.L. (2000). Using Atom Transfer Radical Polymerization To Amplify Monolayers of Initiators Patterned by Microcontact Printing into Polymer Brushes for Pattern Transfer. Macromolecules.

[17] Joubert M., Delaite C., Bourgeat-lami E., Dumas P. (2003). Ring-Opening Polymerization of e-Caprolactone and L-Lactide from Silica Nanoparticles Surface. J. Polym. Sci. Part A Polym. Chem..

[18] Lahann J., Langer R. (2001). Surface-Initiated Ring-Opening Polymerization of e-Caprolactone from a Patterned Poly (hydroxymethyl-p-xylylene). Macromol. Rapid Commun..

[19] Kaiser A., Dutz S., Schmidt A.M. (2009). Kinetic Studies of Surface-Initiated Atom Transfer Radical Polymerization in the Synthesis of Magnetic Fluids. J. Polym. Sci. Part A Polym. Chem..

[20] Choi I.S., Langer R. (2001). Surface-Initiated Polymerization of l-Lactide: Coating of Solid Substrates with a Biodegradable Polymer. Macromolecules.

[21] Clarson S.J., Semlyen J.A. (1993). Siloxane Polymers.

[22] Mark J.E. (2000). Overview of Siloxane Polymers. Am. Chem. Soc..

[23] Normand F., He X.W., Widmaier J.M., Meyer G.C. (1989). Linear polycondensation of a,w-dihydroxy polydimethylsiloxane, catalyzed by stannous octoate. Eur. Pol. J..

[24] He X., Lappa A., Herz J. (1988). Chain branching of poly(dimethylsiloxane): A competitive side reaction of the hydrosilylation reaction. Makromol. Chem..

[25] Wilczek L., Rubinsztajn S., Chojnowski J. (1986). Comparison of the cationic polymerization of octamethylcyclotetrasiloxane and hexamethylcyclotrisiloxane. Makromol. Chem..

[26] Dubois P., Coulembier O., Raquez J.-M. (2009). Handbook of Ring-Opening.

[27] Hurd D.T. (1955). On the Mechanism of the Acid-catalyzed Rearrangement of Siloxane Linkages in Organopolysiloxanes. J. Am. Chem. Soc..

[28] Polymerisation T.A. (1965). The Acid-catalysed Polymerisation of Cyclosiloxanes. Part I. The Kinetics of the Polymerization of Octamethylcycloterasiloxane Catalyzed by anhydrous Ferric Chloride-Hydrogen Chloride. J. Chem. Soc..

[29] Bi C., Xiaoii Z., Lingmint Y.I., Fengqiu C. (2007). Cationic Ring Opening Polymerization of Octamethylcyclotetrailoxane Initiated by Acid Treated Bentonite. Chin. J. Polym. Sci..

[30] Wilczek L., Chojnowski J. (1981). Acidolytic Ring Opening of Cyclic Siloxane and Acetal Monomers. Role of Hydrogen Bonding in Cationic Polymerization Initiated with Protonic Acids. Macromolecules.

[31] Chojnowski J. (1991). Kinetieally Controlled Siloxane Ring-Opening Polymerization. J. Inorg. Organomet. Polym..

[32] Grzelka A., Chojnowski J., Fortuniak W., Richard G., Hupfield P.C. (2004). Kinetics of the Anionic Ring Opening Polymerization of Cyclosiloxanes Initiated with a Superbase. J. Inorg. Organomet. Polym..

[33] Semlyen J.A. (1976). Ring-Chain Equilibria and the Conformations of Polymer Chains. Adv. Pol. Sci..

[34] Hurd B.D.T., Osthoff R.C., Corrin M.L., Si S.O., Osthoff C. (1954). The Mechanism of the Base-catalyzed Rearrangement of Organopolysiloxanes. J. Am. Chem. Soc..

[35] Chojnowski J., Rubinsztajn S., Fortuniak W., Kurjata J. (2007). Oligomer and polymer formation in hexamethylcyclotrisiloxane (D 3)-Hydrosilane systems under catalysis by tris(pentafluorophenyl) borane. J. Inorg. Organomet. Polym..

[36] Fuchise K., Sato K., Shimada S., Igarashi M., Sato K., Shimada S. (2018). Organocatalytic controlled/living ring-opening polymerization of cyclotrisiloxanes initiated by water with strong organic base catalysts†. R. Soc. Chem..

[37] Molenberg A., Moller M. (1997). Polymerization of cyclotrisiloxanes by organolithium compounds and P2-Et base. Macromol. Chem. Phys..

[38] Hupfield P.C., Taylor R.G. (1999). Ring-Opening Polymerization of Siloxanes Using Phosphazene Base Catalysts. J. Inorg. Organomet. Polym..

[39] Molenberg A., Moller M. (1995). A fast catalyst system for the ring-opening polymerization of cyclosiloxanes. Macromol. Rapid Commun..

[40] Esswein B., Molenberg A., Möller M. (1996). Use of polyiminophosphazene bases for ring-opening polymerizations. Macromol. Symp..

[41] Schwesinger R., Schlemper H. (1987). Peralkylated Polyaminophosphazenes-Extremely Strong, Neutral Nitrogen Bases. Angew. Chem. Int. Ed..

[42] Schwesinger B.R., Mij M., Peters K., Von Schnering H.G. (1987). Novel, Very Strongly Basic, Pentacyclic “Proton Sponges” with Vinamidine Structure**. Angew. Chem. Int. Ed..

[43] Esswein B., Möller M. (1996). Polymerization of Ethylene Oxide with Alkyllithium Compounds and the Phosphazene Base “tBu-P4”. Angew. Chem. Int. Ed..

[44] Wagner H.L. (1985). The Mark-Houwink-Sakurada Equation for the Viscosity of Atactic Polystyrene. J. Phys. Chem. Ref. Data.

[45] Mark James E. (1999). Polymer Data Handbook.

[46] Hartlen K.D., Athanasopoulos A.P.T., Kitaev V. (2008). Facile Preparation of Highly Monodisperse Small Silica Spheres (15 to >200 nm ) Suitable for Colloidal Templating and Formation of Ordered Arrays. Langmuir.

[47] Ozaki M., Kratohvil S., Matjevic E., Matijević E. (1984). Formation of Monodispersed Spindle-Type Hematite Particles 1. J. Colloid Interface Sci..

[48] Wagner J., Autenrieth T., Hempelmann R. (2002). Core shell particles consisting of cobalt ferrite and silica as model ferrofluids [CoFe_2_O_4_-SiO_2_ core shell particles]. J. Magn. Magn. Mater..

[49] Van Ewijk G.A., Vroege G.J., Philipse A.P. (1999). Convenient preparation methods for magnetic colloids. J. Magn. Magn. Mater..

[50] Van Dyke M.E., Clarson S.J. (1998). Reaction Kinetics for the Anionic Ring-Opening Polymerization of Tetraphenyltetramethylcyclotetrasiloxane Using a Fast Initiator System. J. Inorg. Organomet. Polym..

[51] Zhao J., Hadjichristidis N., Schlaad H. (2015). Polymerization Using Phosphazene Bases. Anionic Polymerization.

[52] Jacobson H., Stockmayer W.H. (1950). Intramolecular Reaction in Polycondensations. I. The Theory of Linear Systems. J. Chem. Phys..

[53] Flory P.J., Semlyen J.A. (1966). Macrocyclization Equilibrium Constants and the Statistical Configuration of Poly(dimethylsiloxane) Chains. J. Am. Chem. Soc..

[54] Hubert S., Hemery P., Boileau S. (1986). Anionic polymerization of cyclosiloxanes with cryptates as counterions: New results. Makromolekulare Chemie. Macromolecular Symposia.

[55] Hinman J.G., Lough A.J., Morris R.H. (2007). Properties of the Polyhydride Anions [WH5(PMe2Ph)3] and [ReH4(PMePh2)3] and Periodic Trends in the Acidity of Polyhydride Complexes. Inorg. Chem..

[56] Pibre G., Chaumont P., Fleury E., Cassagnau P. (2008). Ring-opening polymerization of decamethylcyclopentasiloxane initiated by a superbase: Kinetics and rheology. Polymer.

[57] Ek S., Root A., Peussa M., Niinistö L. (2001). Determination of the hydroxyl group content in silica by thermogravimetry and a comparison with 1H MAS NMR results. Thermochim. Acta.

