# Supplementary material for: Creation of a PDMS Polymer Brush on SiO2-Based Nanoparticles by Surface-Initiated Ring-Opening Polymerization
Source: Polymers (Basel). 2020 Apr 2;12(4):787. doi: 10.3390/polym12040787 (PMC7240505; doi:10.3390/polym12040787)
Supplement: Supplementary file 1 [file polymers-12-00787-s001.pdf]

Electronic Supplementary Information

Creation of a PDMS polymer brush on SiO_2 based nanoparticles by surface-initiated ring opening polymerization

Karin Koch,[1] Sven Geller,[1] Kubilay Acar,[1] Patricia Bach,[1] Ekaterina Tsarenko,[1] and Annette Schmidt[1]*

[1] Universität zu Köln, Department Chemie, Institut für Physikalische Chemie; annette.schmidt@uni-koeln.de

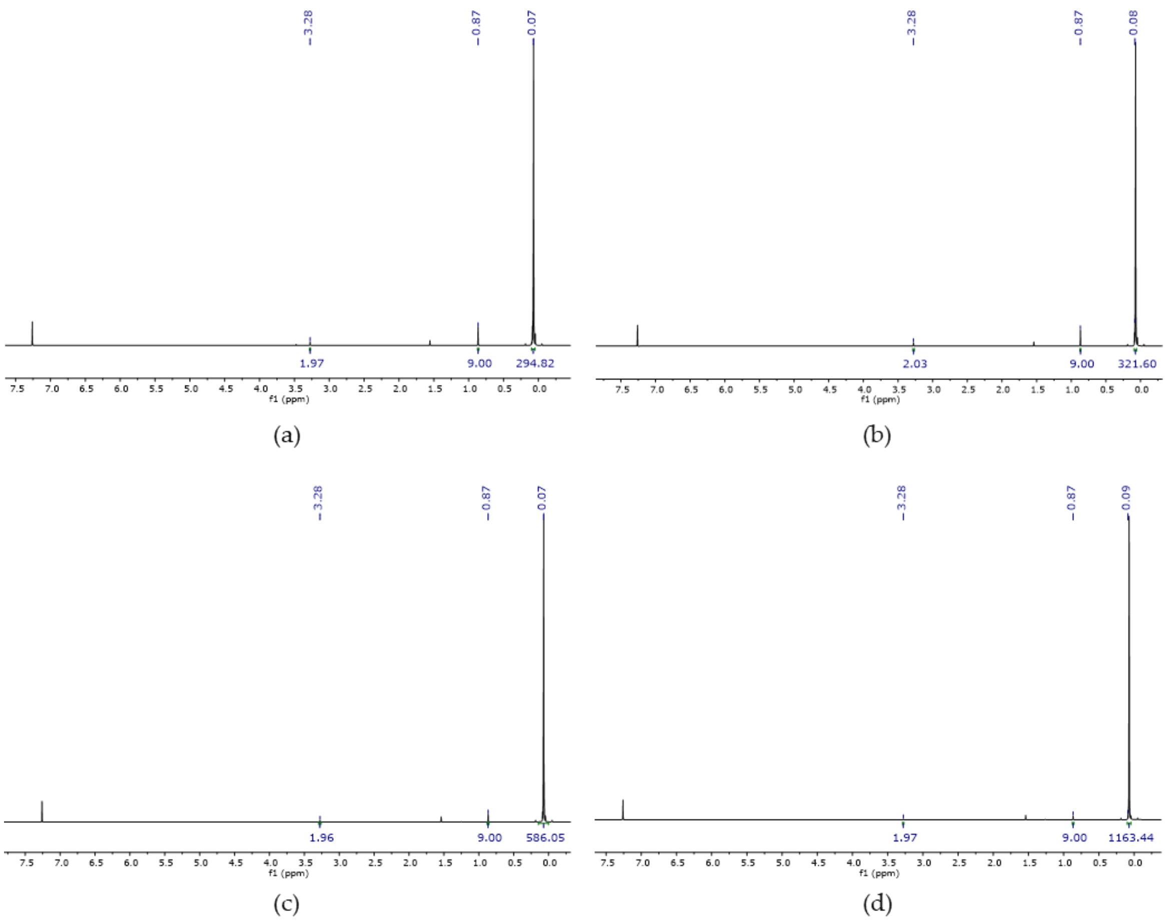

Figure S1. ^{1}H-NMR spectra measured in $CDCl_3$ of ROP of M_4 performed in toluene with NeOH as initiator and P_4-*t*-Bu as catalyst, for an monomer-to-initiator ratio of a) $n_M/n_I = 5$, b) $n_M/n_I = 10$, c) $n_M/n_I = 25$ and d) $n_M/n_I = 50$.

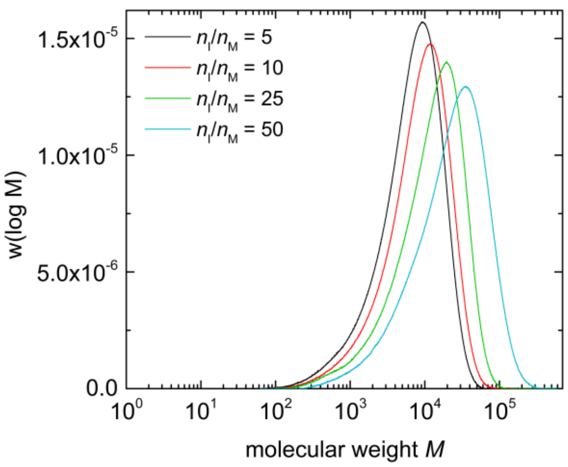

Figure S2. Molar mass distribution of ROP of M_4 in toluene with NeOH as initiator, P_4-*t*-Bu as catalyst and different monomer-to-initiator ratios measured by SEC.

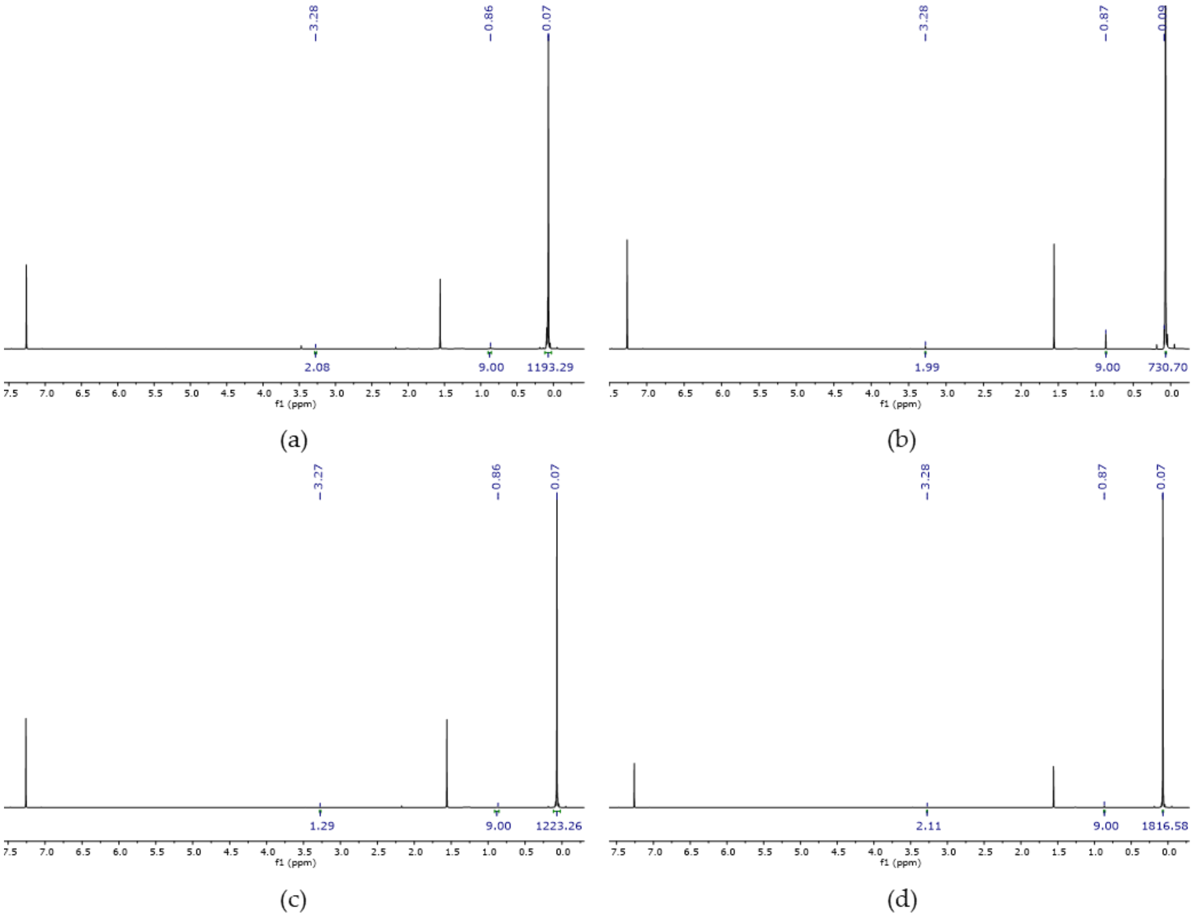

Figure S3. ^{1}H-NMR spectra measured in $CDCl_3$ of ROP of M_4 in toluene with NeOH as initiator, P_2-*t*-Bu as catalyst for an monomer-to-initiator ratio of a) $n_M/n_I = 5$, b) $n_M/n_I = 10$, c) $n_M/n_I = 25$ and d) $n_M/n_I = 50$.

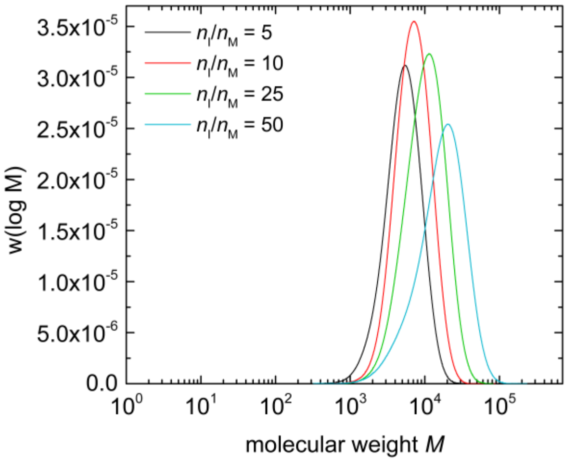

Figure S4. Molar mass distribution of ROP of M_4 in toluene with NeOH as initiator, P_2-*t*-Bu as catalyst and different monomer-to-initiator ratios measured by SEC.

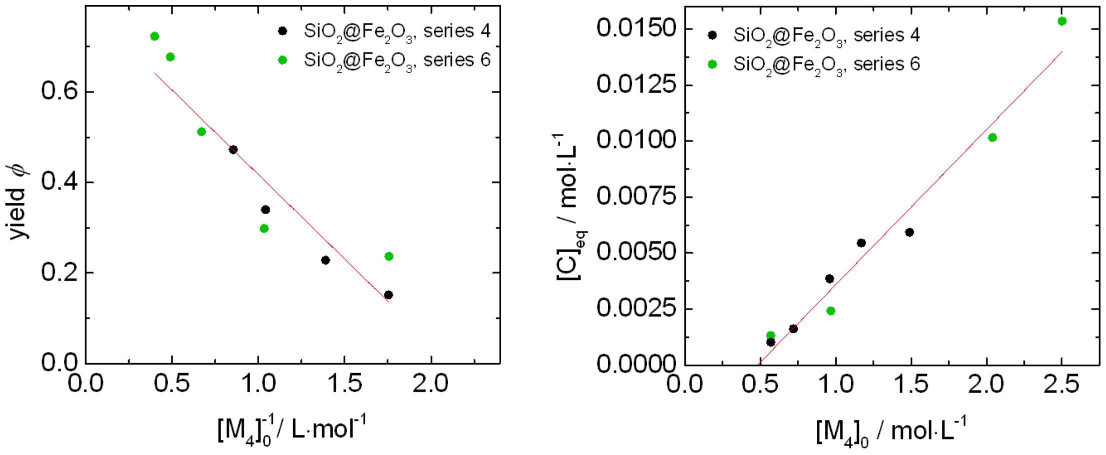

(a) (b)

Figure S 5. a) Yield as a function of the reciprocal monomer concentration and b) polymer concentration from SI-ROP of M_4 with $SiO_2@Fe_2O_3$ particles as a function of $[M_4]_0$ for series 4 and series 6.

Table S1. Results of elemental analysis for SiO_2 and $SiO_2@\alpha$-Fe_2O_3.

**sample**	**N [%]**	**C [%]**	**H [%]**
SiO_2	0.00	0.02	2.31
$SiO_2@\alpha$-Fe_2O_3	0.00	0.03	2.66

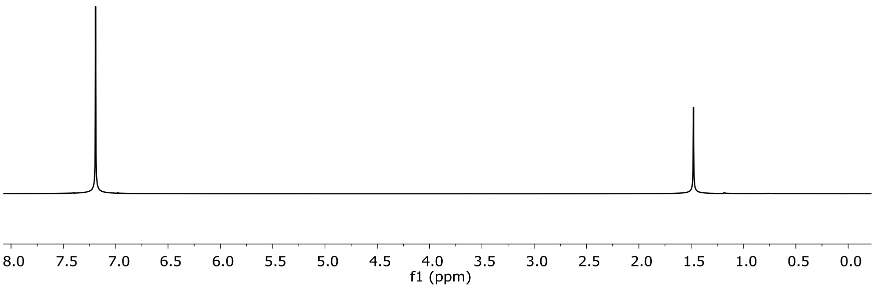

Figure S 6. ^{1}H-NMR spectrum of SiO_2 particles measured in $CDCl_3$. The spectrum only shows the solvent related peaks.

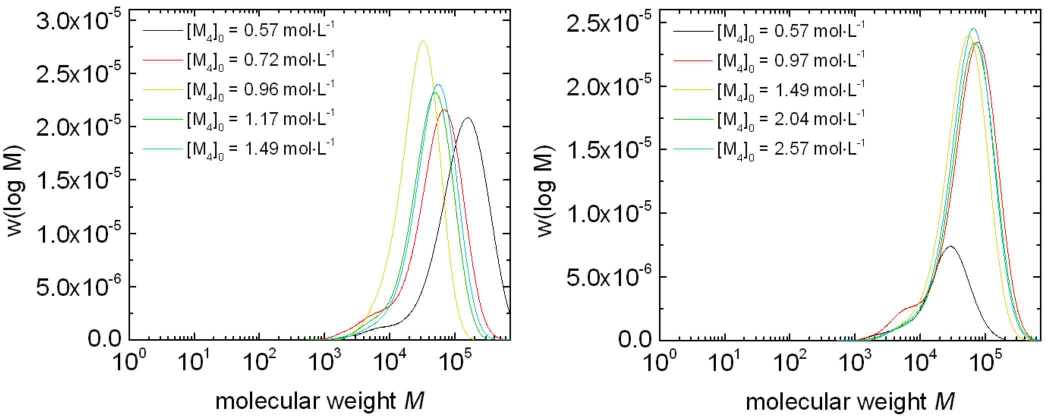

(a) (b)

Figure S7. Molar mass distribution of free Polymer obtained during SI-ROP of M_4 with SiO_2 particles as a function of $[M_4]_0$ for a) *series 3* and b) *series 5*.

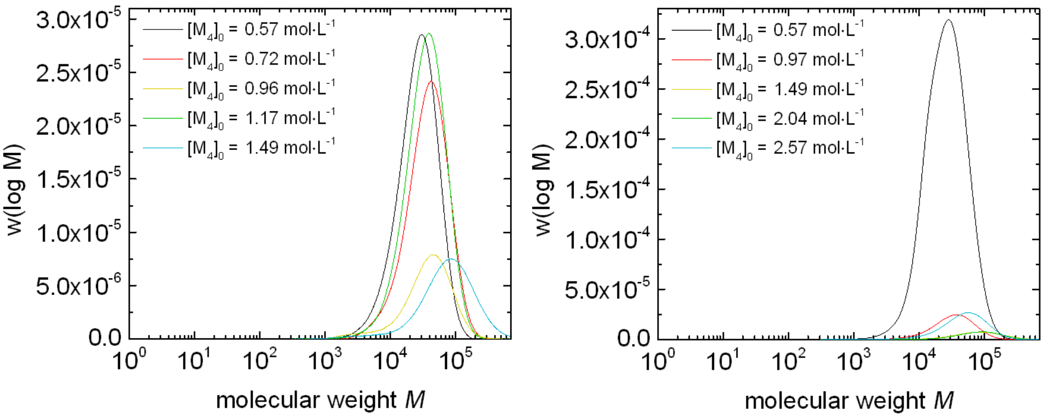

(a) (b)

Figure S8. Molar mass distribution of free PDMS obtained during the SI-ROP of M_4 with $SiO_2@Fe_2O_3$ particles as a function of $[M_4]_0$ for a) *series 4* and b) *series 6*.

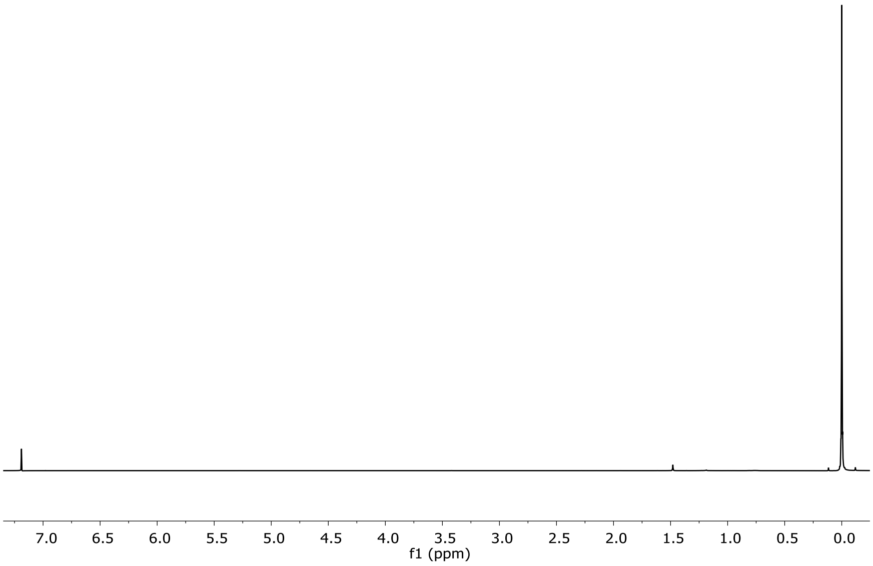

Figure S9. Example for ^{1}H-NMR spectrum of free PDMS obtained during the SI-ROP of M_4 with SiO_2 particles measured in $CDCl_3$. Only the TMS signal from PDMS and the signals for the solvent can be observed. The spectra of all free polymer fractions obtained in series 3 – 6 show analogous results.

The yield regarding to surface-attached PDMS and free PDMS for the different injection methods are listed in Table S2. Whereas surface attached PDMS yield Φ_P decreases with dropwise addition of M_4 compared to the samples prepared by the common method with the same no significant change for the free PDMS is observed. By further addition of M_4 after 2 h Φ_P remains constant, because part of the added monomer is added to the polymer brushes at the particle surface. However, the yield for the free PDMS decreases and its molar mass only slightly increases compared with the amount of added monomer. This indicates that only a small amount of M_4 reacts with the free polymer chains.

Table S2. Yield of surface-attached PDMS and free PDMS for the SI-ROP of M_4 performed with different injection methods.

**sample**	**injection**	Φ_P %	Φ_{free} %	$M_{n,free}$ g·mol^{-1}
SiO_2	common	1.5	62.3	35748
SiO_2	adding more M_4 after 2 h	1.7	29.7	46321
SiO_2	dropwise	1.1	69.2	41318
$SiO_2@\alpha$-Fe_2O_3	common	1.6	66.0	37084
$SiO_2@\alpha$-Fe_2O_3	adding more M_4 after 2 h	1.5	25	48652
$SiO_2@\alpha$-Fe_2O_3	dropwise	0.8	68.2	27493

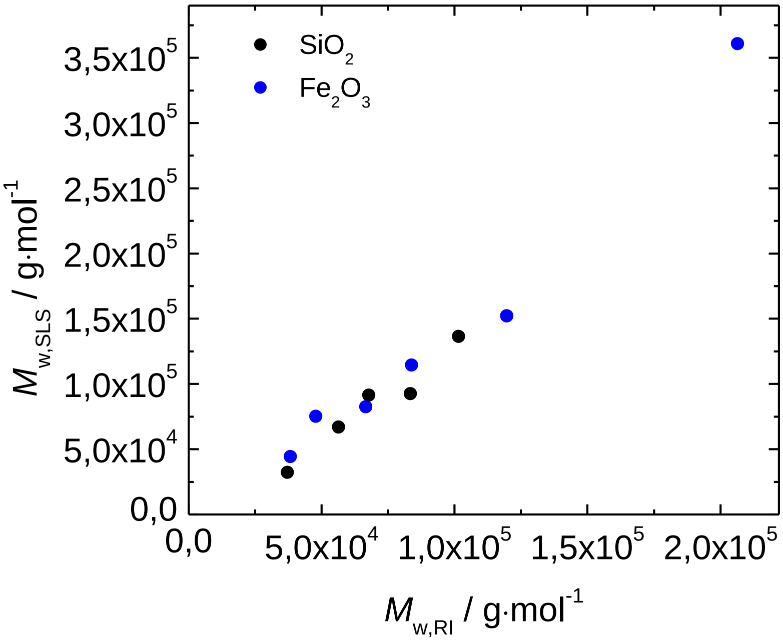

Figure S10. Correlation between the mass-average molar mass obtained by conventional calibration and Mark-Houwing correction (Mw,RI) and the absolute result (Mw, SLS) for the free polymer fractions obtained in series 3 – 6. The slope of the linear correlation is 1.40.

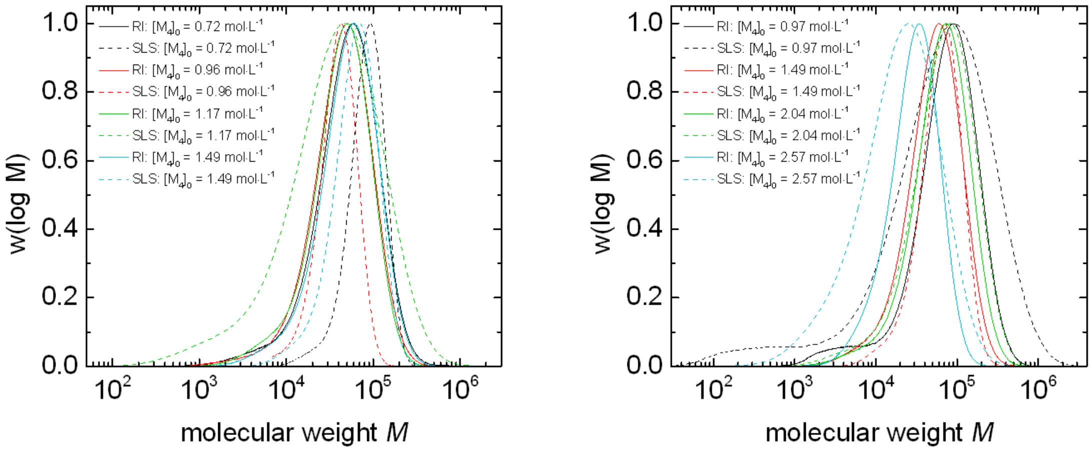

(a) (b)

Figure S 111. Molar mass distribution measured with RI and SLS detector of free Polymer obtained during SI-ROP of M4 with SiO_2 particles as a function of $[M_4]_0$ for a) *series 3* and b) *series 5*. The curves are normalized to 1 to compare the data obtained by SLS and RI.

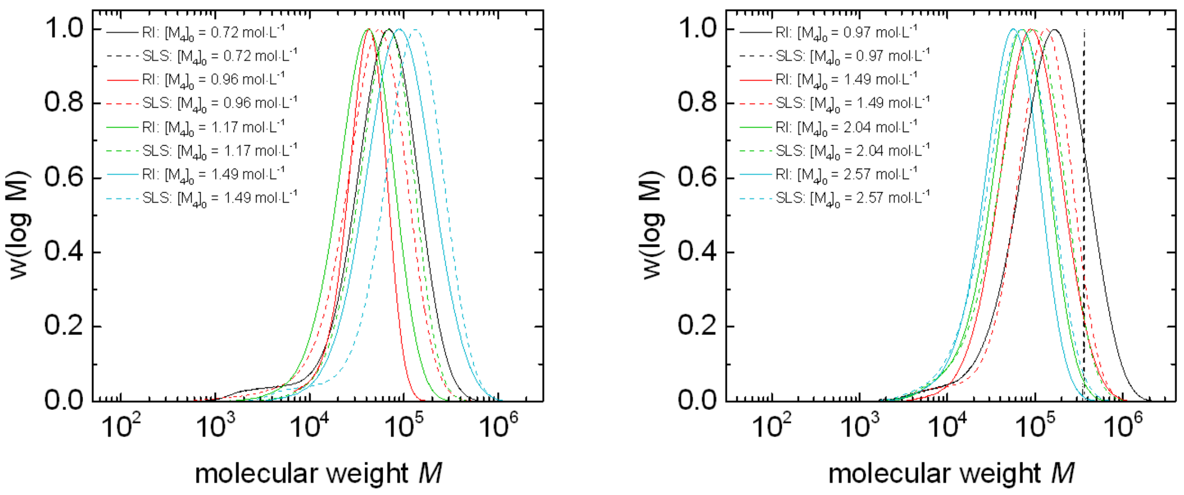

(a) (b)

Figure S12 Molar mass distribution measured with RI and SLS detector of free polymer obtained during SI-ROP of M_4 with Fe_2O_3 particles as a function of $[M_4]_0$ for a) *series 4* and b) *series 6*. The curves are normalized to 1 to compare the data obtained by SLS and RI.
